# High ecological resilience of the sea fan *Gorgonia ventalina* during two severe hurricanes

**DOI:** 10.7717/peerj.10315

**Published:** 2020-11-11

**Authors:** Peter J. Edmunds

**Affiliations:** Department of Biology, California State University, Northridge, CA, United States of America

**Keywords:** Octocorals, Gorgonians, Caribbean, Coral reef, Virgin Islands

## Abstract

Since about the turn of the millennium, octocorals have been increasing in abundance on Caribbean reefs. The mechanisms underlying this trend have not been resolved, but the emergent species assemblage appears to be more resilient than the scleractinians they are replacing. The sea fan *Gorgonia ventalina* is an iconic species in the contemporary octocoral fauna, and here its population dynamics are described from St. John, US Virgin Islands, from 2013 to 2019. Mean densities of *G. ventalina* at Yawzi Point (9-m depth) varied from 1.4–1.5 colonies m^−2^, and their mean heights from 24–30 cm; nearby at Tektite (14-m depth), they varied from 0.6–0.8 colonies m^−2^ and from 25–33 cm. These reefs were impacted by two Category 5 hurricanes in 2017, but neither the density of *G. ventalina*, the density of their recruits (< 5-cm tall), nor the height of colonies, differed among years, although growth was depressed after the hurricanes. Nevertheless, at Tektite, colony height trended upwards over time, in part because colonies 10.1–20 cm tall were reduced in abundance after the hurricanes. These trends were sustained without density-associated effects mediating recruitment or self-thinning of adults. The dynamics of *G. ventalina* over seven years reveals the high resilience of this species that will contribute to the persistence of octocorals as a dominant state on Caribbean reefs.

## Introduction

Tropical coral reefs are undergoing profound changes in community structure as scleractinians are dying (Jackson et al., 2014; Edmunds & Lasker, 2016; Hughes et al., 2018), thus transitioning alternative taxa into functional dominance (Norström et al., 2009). These changes are being accompanied by modifications in the ecological processes structuring reef communities (Jouffray et al., 2015; Hughes et al., 2018), as well as the goods and services they provide (Wild et al., 2011), for instance, through depressed community calcification (Eyre et al., 2018), and impaired capacity to protect shorelines from erosion (Elliff & Silva, 2017). These trends highlight the need to study the ecology of emerging tropical reef communities, and on shallow Caribbean reefs, one front of this effort focuses on octocorals (Ruzicka et al., 2013; Lasker et al., 2020).

Octocorals have long been recognized as important on Caribbean reefs (Cary, 1914; Kinzie, 1973), but over the last 20 years, their importance has been accentuated by regional increases in their abundance (Ruzicka et al., 2013; Lenz et al., 2015; Lasker et al., 2020). The causes of this trend remain unclear, but rapid vertical growth (Borgstein, Beltrán & Prada, 2020) is advantageous in allowing many octocorals to escape the risks of competition with spatially aggressive benthic taxa. Competition with macroalgae, for example, is detrimental for small scleractinians (Box & Mumby, 2007), whereas arborescent octocorals can quickly extend above macroalgae from a small site of attachment (Jackson, 1979; Borgstein, Beltrán & Prada, 2020). The positive demographic implications of rapid growth are augmented by recruitment, which is modest relative to scleractinians when octocoral recruits are considered as colonies ≤ 10 cm tall (Lasker et al., 2020), but can be high when measured as polyps on settlement tiles evaluated over days-weeks (Wells et al., in review). Together, these features are likely to be important in supporting greater ecological resilience of octocorals relative to scleractinians (Tsounis & Edmunds, 2017), and promoting octocoral persistence to form structurally dominant communities on contemporary reefs (Lasker et al., 2020).

Central to understanding the mechanisms through which octocorals have increased in abundance on Caribbean reefs is their demography. However, after decades of neglect, demographic data are available for only a few octocorals (e.g., Lasker, 1991; Yoshioka, 1994; Bruno et al., 2011). These data reveal situations under which octocorals are more resistant to environmental challenges than scleractinians, such as when reefs are affected by high temperature (Goulet et al., 2017), and during recovery following storms (Tsounis & Edmunds, 2017; Lasker et al., 2020). In warm seawater, octocorals are less likely than scleractinians to bleach (Prada, Weil & Yoshioka, 2010; Goulet et al., 2017), and during severe wave events, octocorals tend to be lost from the reef through failure of the substratum to which they are attached, rather than breakage of the colony (Kinzie, 1973; Birkeland, 1974). Addressing mechanisms promoting octocoral success has benefitted from the analogy with terrestrial forests (Rossi et al., 2017), which has motivated consideration of the emergent consequences of dense stands of octocorals. These include creating a unique sub-canopy flow regime (Guizien & Ghisalberti, 2016), modified recruitment (Privitera-Johnson, Lenz & Edmunds, 2015), and a context in which self-thinning (sensu Yoda et al., 1963) might play a role in regulating population size (Edmunds & Lasker, 2019).

Sea fans in the genus *Gorgonia* are iconic on Caribbean reefs and have been studied for decades. Early work focused on colony orientation relative to seawater flow (Wainwright & Dillon, 1969), predation by the gastropod *Cyphoma gibbosum* (Birkeland & Gregory, 1975), natural products chemistry (Ciereszko & Karns, 1973), and population biology (Birkeland, 1974). Arguably, however, *Gorgonia* spp. is best known through its infection with a disease that first was reported in 1984 from Costa Rica (Guzmán & Cortés, 1984), and later attributed to the fungus, *Aspergillus sydowii* (Smith et al., 1996). Aspergillosus has negatively affected populations of *G. ventalina* and *G. flabellum* throughout the Caribbean (Kim & Rypien, 2016), and has motivated one of the most comprehensive demographic analyses of any octocoral (Bruno et al., 2011).

The present study describes the population dynamics of *Gorgonia ventalina* from 2013–2019 at two sites on the south shore of St. John, US Virgin Islands, and uses the results to explore population stability over a period punctuated by severe storms. Stands of *G. ventalina* at Yawzi Point (9-m depth) and Tektite (14-m depth) are ∼650 m apart, and occur as dense aggregates on a framework dominated by the scleractinian *Orbicella annularis* (Edmunds, 2015). Since *G. ventalina* reproduces through gonochoric spawning (Petes et al., 2003) with pelagic larvae that disperse over <2 km (Andras, Rypien & Harvell, 2012), the two stands are likely to function as a single population. The octocoral communities at these sites are less well developed than those found a few hundred meters away (Tsounis et al., 2018; Lasker et al., 2020), and are characterized by high relative abundance of *G. ventalina* (i.e., 61–66% of colonies were *G. ventalina* in 2019). With high densities of one species, it is easier to make inferences regarding the demographic properties mediating population dynamics, because intraspecific interactions are likely to be more important than interspecific interactions.

The main objective of this study was to test for changes in the *G. ventalina* stands over 7 years that included two Category 5 hurricanes in September 2017 (Edmunds, 2019a). Using the results from the main objective as a context, changes in the stands of *G. ventalina* were explored for signs that they conformed to the changes expected from underlying density-dependent mechanisms (recruitment and self-thinning (Yoda et al., 1963; Westoby, 1984)) that can regulate population sizes of plants and animals (Westoby, 1984; Fréchette & Lefaivre, 1995). These mechanisms appear to modulate the population size of some octocorals (Privitera-Johnson, Lenz & Edmunds, 2015; Linares et al., 2008; Edmunds & Lasker, 2019), but exploring for population regulation in species assemblages is problematic, especially for self-thinning (White, 1981). By studying *G. ventalina* at two sites in St. John, it was possible to reduce these effects and use this system to explore: (1) the association between recruitment and density of adult colonies (i.e., density-dependent recruitment), and (2) the association between biomass (using colony height as a proxy) and density (i.e., self-thinning). Interpretation of the trends revealed by these analyses is subject to several caveats, notably that the association between abundance and other variables cannot demonstrate density-dependence (i.e., cause and effect), and the experimental design has limitations. These include the range of densities of *G. ventalina* that were sampled, and the necessity of pooling results among years to explore density-associated affects. These caveats are addressed in the discussion.

## Materials and Methods

The study focused on *Gorgonia ventalina* at Yawzi Point and Tektite within Great Lameshur Bay, and the research was completed under permits issued by the Virgin Islands National Park (number VIIS-2013-SCI-0015). These reefs have been monitored from 1987 to present (Edmunds, 2015; Edmunds, 2019a; Edmunds, 2019b), and the present analysis was superimposed on the same study areas. At each site, three permanent transects were installed in 1987, with each ∼10 m long, parallel to one another, and ∼5 m apart. Contiguous photoquadrats (1 × 1 m) have been recorded along each transect to quantify the cover of benthic taxa.

**Figure 1:**
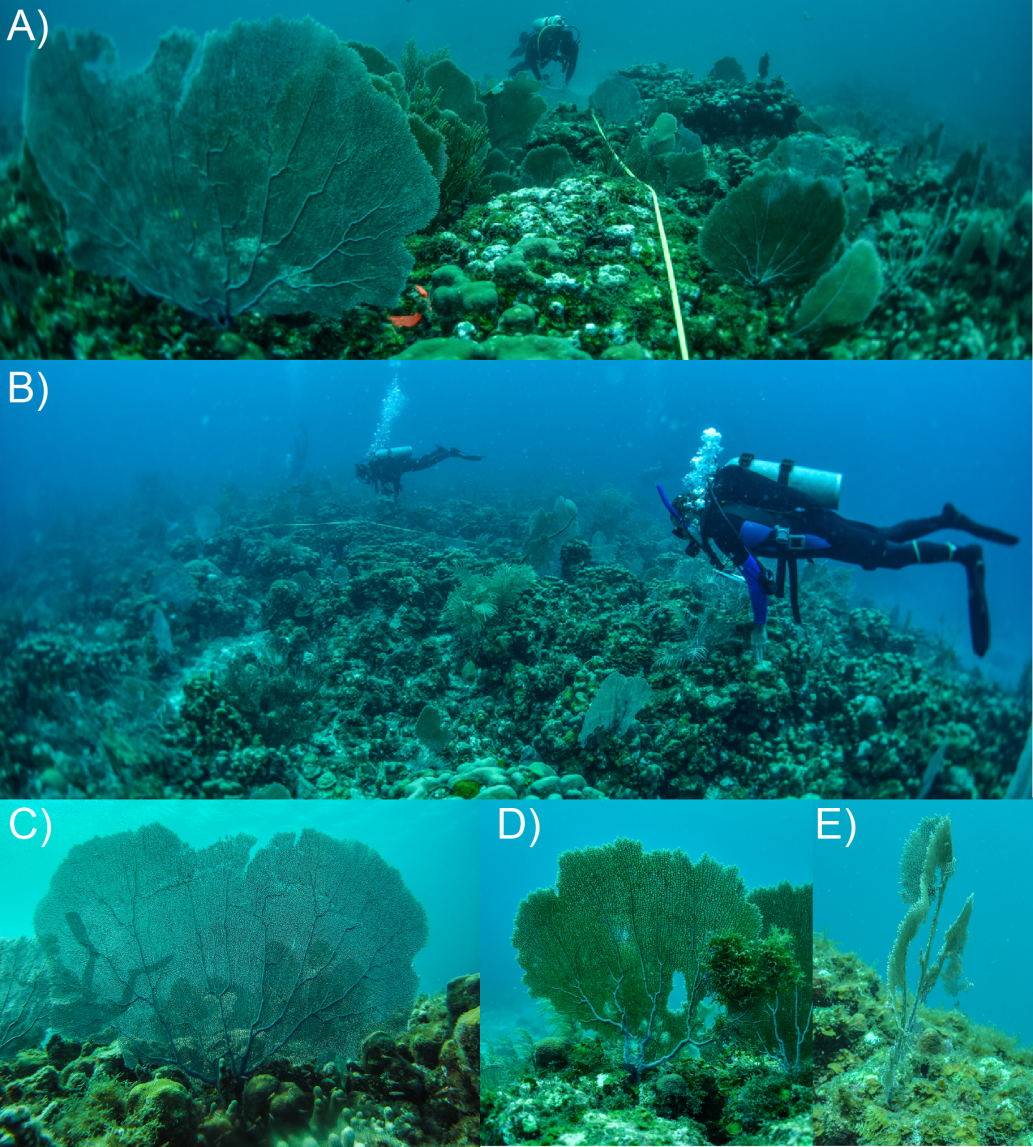
Study sites at Yawzi Point (9-m depth) (A) and Tektite (14-m depth) (B) in 2016. Pictures (A, B) show high abundance of *Gorgonia ventalina*, with representative colonies below: (C) symmetric fan with no mortality, (D) asymmetric fan with small amounts of mortality, and (E) ragged fan with extensive partial mortality. Ragged fans accounted for 10–12% of fans over the 7-year study, but by year, they accounted for 0% (Yawzi Point in 2015) to 24% (Tektite in 2018) of fans. Photo credits: P.J. Edmunds.

Starting in August 2013, colonies of *G. ventalina* ± 1 m of each transect were surveyed, with their sizes recorded as height. Each colony was mapped through Cartesian coordinates along each transect, and their height was recorded using a flexible tape measure (± 0.5 cm) as the linear distance from the holdfast to the highest distal portion of living tissue. Fans that were symmetrical or slightly imperfect in shape with small areas of mortality (Figs. 1C, 1D) were easily measured, but fans that were torn and were affected by partial mortality (i.e., categorized as “ragged” fans, Fig. 1E) posed challenges for measurement. The size of ragged fans was recorded as their greatest height, which overestimated their size relative to the amount of *G. ventalina* tissue. Field logistics prevented finer resolution of tissue area (e.g., through photography (e.g., Bruno et al., 2011)), but separate analyses of photoquadrats were used to evaluate the abundance of ragged colonies in each year.

The surveys of *G. ventalina* were repeated annually in August from 2013 to 2019, and on each occasion colonies were mapped and their sizes recorded as above. Sizes and Cartesian coordinates were compared between consecutive years to identify colonies that were evaluated in both years, colonies that had died between years (lost from the reef or reduced to a horny axis without tissue), or small colonies that recruited between years. To quantify the number of colonies that were ragged, photoquadrats along each line were used to evaluate the number of colonies that were: (a) symmetrical or asymmetrical with partial mortality, or (b) ragged (Figs. 1C–1D). Colonies were assigned to these categories in planar view, and were based on surveys of half the reef area compared to the areas surveyed for *G. ventalina* in situ (i.e., photoquadrats were recorded along one side of the transects).

### Analyses

Mean densities of *G. ventalina* were compared among times using repeated measures ANOVA in which transects were repeatedly surveyed over time; a non-parametric Friedman test was used when statistical assumptions were not met. Mean colony sizes were compared over time using one-way ANOVA in which each colony was a replicate. The density of *G. ventalina* recruits was evaluated from colonies ≤ five cm tall (after Yoshioka, 1996), and their densities and heights were compared over time as described above. Colonies were considered to have left the recruiting size class when they were >five cm tall. The percentage distribution of colonies among sizes classes over time was evaluated using size (height) classes of 10 cm, with the largest class including colonies between 60 and 100 cm tall. The distribution of colonies among size classes was tested for variation among years using *χ*^2^ contingency tables. Growth was recorded as the change in height between consecutive years for colonies that were located in both years, and growth was compared over time using non-parametric Kruskal-Wallis (three or more groups) or Mann–Whitney U-Tests (two groups).

Density-associated effects were explored through analyses of the relationships between mean height and mean density for self-thinning, and between mean density of recruits and mean density of colonies > five cm tall, using transects as statistical replicates. Evidence for self-thinning would be revealed by an inverse relationship size and density, and a slope of the log size versus log density relationship of ∼1.5 (Westoby, 1984). Evidence of density-associated recruitment would be revealed by a linear relationship between the density of recruits and larger colonies, and a linear relationship (either positive or negative) between per-capita recruitment and density of colonies >five cm tall. Per capita recruitment was calculated by dividing the density of recruits by the density of colonies >five cm tall.

## Results

The study reefs were dominated by dead colonies of *Orbicella annularis* (Yawzi Point), or live colonies of the same species (Tektite), and on these surfaces, *Gorgonia ventalina* were common, conspicuous, and often large (Fig. 1). Colonies at both sites were separated from one another on a scale of centimeters-to-a-meter, and while most colonies were symmetrical without mortality, or asymmetrical with small areas of partial mortality, some were ragged with little tissue and large tears and omissions in the fan surface (Figs. 1C–1D). Overall (pooled among years), 10% of colonies were ragged at Yawzi Point, and 12% at Tektite, and the mean percentage of ragged colonies increased from 7 ± 9% before the storms to 20 ± 1% after the storms at Yawzi Point, and from 10 ± 5% to 16 ± 12% at Tektite (± SD, *n* = 5 and 2, respectively).

**Figure 2:**
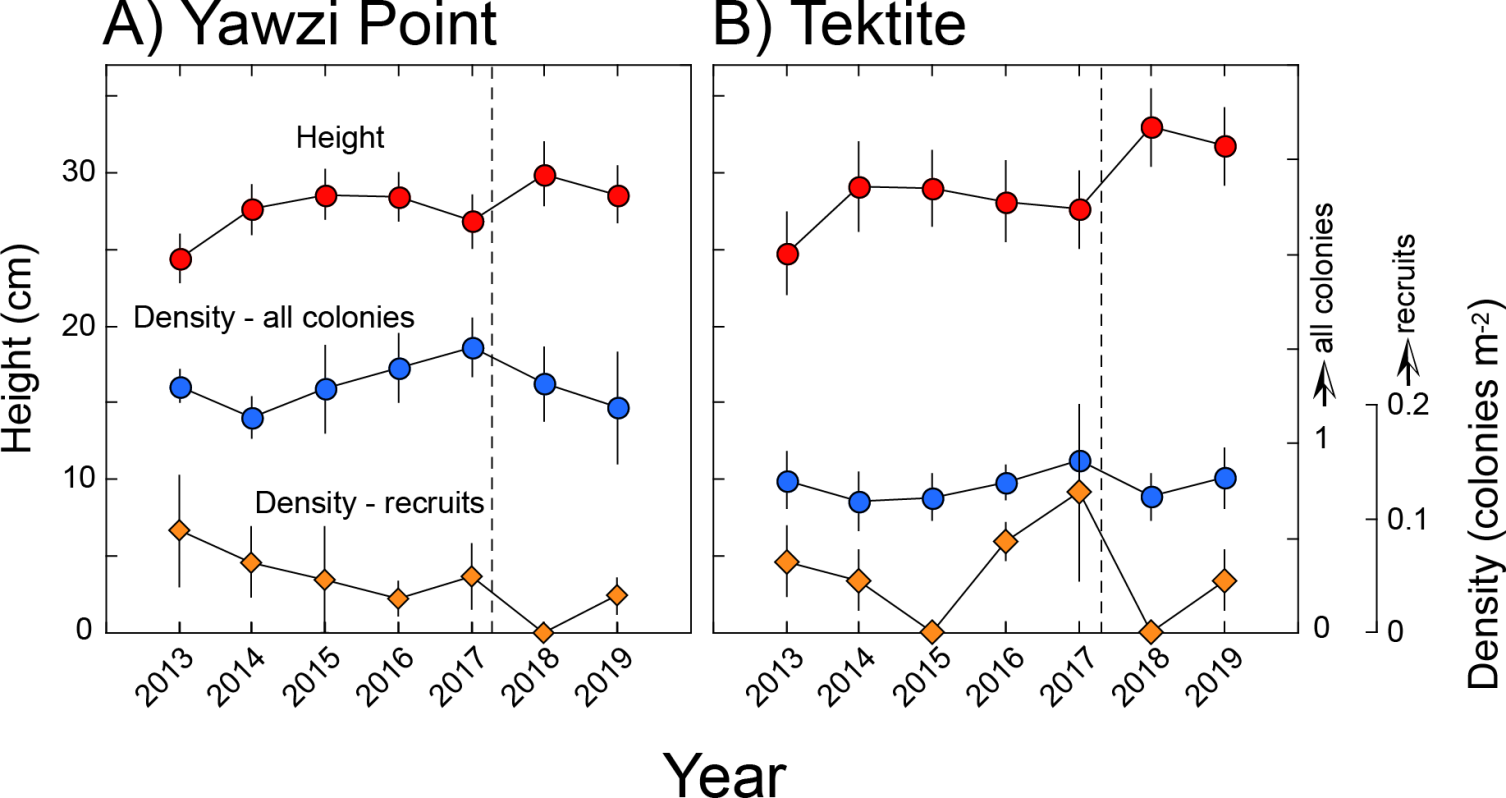
Height and density of *Gorgonia ventalina* at Yawzi Point (A) and Tektite (B). Mean ± SE shown with *n* = 42–97 for height, and *n* = 3 for density. Vertical dashed line shows the impacts of Hurricanes Irma and Maria.

At Yawzi Point, mean (± SE, *n* = 3) densities ranged from 1.41 ± 0.11 colonies m^−2^ in 2014 to 1.51 ± 0.15 colonies m^−2^ in 2017, and mean (± SE) height varied from 24 ± 2 cm in 2013 (*n* = 84) to 30 ± 2 cm in 2018 (*n* = 54). Mean (± SE, *n* = 3) densities of recruits (colonies ≤ five cm) ranged from 0 colonies m^−2^ in 2018, to 0.09 ± 0.05 colonies m^−2^ in 2013 (Fig. 2). Differences over time were not significant for density of all colonies (RM ANOVA, *F* = 2.64, *df* = 5, 10, *p* = 0.140 [2018 excluded since only two transects were surveyed]), heights of all colonies (*F* = 1.101, *df* = 6, 548, *p* = 0.418), and density of juvenile colonies (*χ*^2^ = 1.044, *df* = 5, *p* = 0.959 [2018 excluded since only two transects were surveyed]). Mean density and height of colonies was not linearly related to time (*F* ≤ 4.205, *df* = 1, 5, *p* > 0.096), but the density of recruits declined (*F* = 3.006, *df* = 1, 5, *p* = 0.030).

At Tektite, mean (± SE, *n* = 3) densities ranged from 0.64 ± 0.17 colonies m^−2^ in 2018 to 0.81 ± 0.15 in 2013, and mean (± SE) height varied from 25 ± 3 cm in 2013 (*n* = 53) to 33 ± 3 cm (*n* = 42) in 2018; mean (± SE, *n* = 3) densities of recruits (colonies ≤ five cm) ranged from 0 colonies m^−2^ in 2015, to 0.12 ± 0.08 colonies m^−2^ in 2017 (Fig. 2). Differences over time were not significant for density of all colonies (RM ANOVA, *F* = 1.780, *df* = 6, 12, *p* = 0.186) heights of all colonies (*F* = 1.038, *df* = 6, 345, *p* = 0.400), and density of juvenile colonies (*χ* ^2^ = 8.953, *df* = 6, *p* = 0.176). Mean density of all colonies and recruits was not linearly related to time (*F* ≤ 0.430, *df* = 1, 5, *p* ≥ 0.541), but the height of colonies increased (*F* = 7.809, *df* = 1, 5, *p* = 0.038).

**Figure 3:**
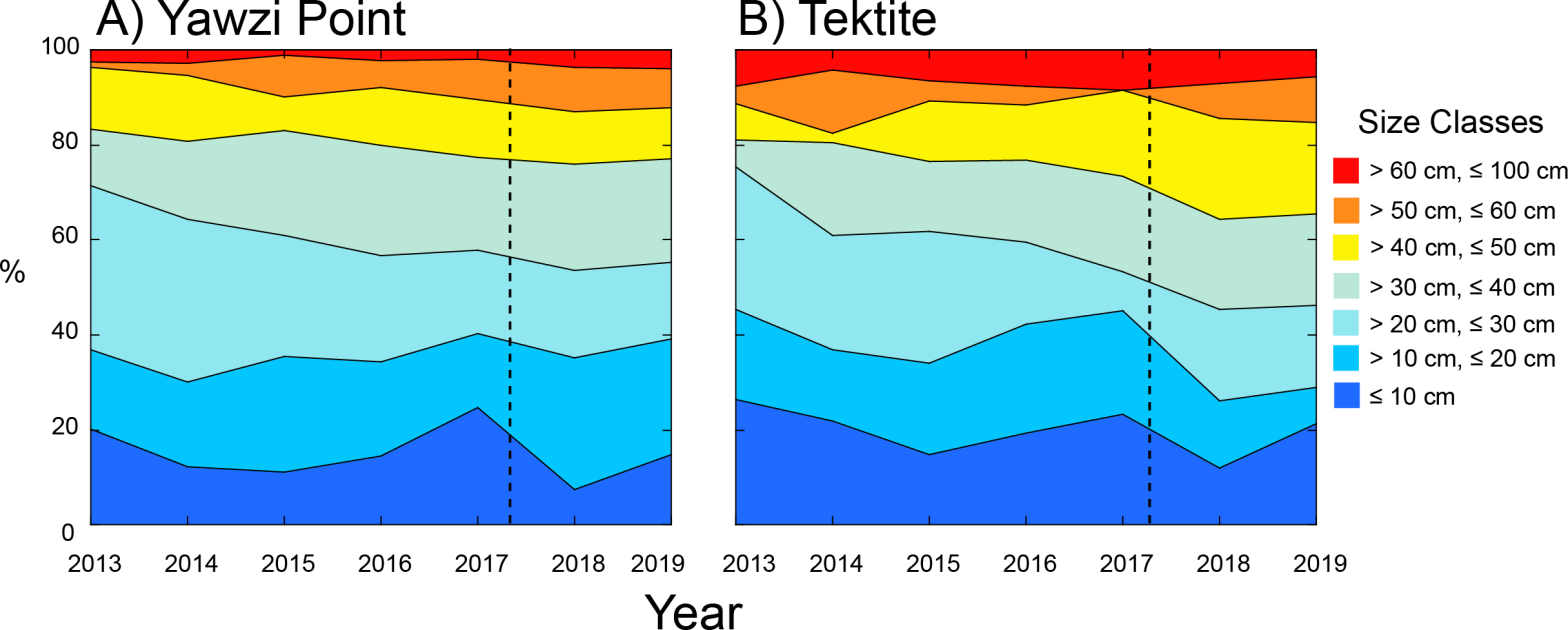
Percentage contribution of colonies of *Gorgonia ventalina* by size class to the populations at Yawzi Point (A) and Tektite (B) from 2013–2019. *n* = 54–97 colonies y^−1^ at Yawzi Point, and *n* = 42–60 colonies y^−1^ at Tektite. Vertical dashed line shows the impacts of Hurricanes Irma and Maria.

The distribution of colonies among size classes revealed that the majority (≥ 54%) was between 10.1 and 40 cm in height at both sites (Fig. 3). The distribution among size classes slightly varied among years with fewer of the smallest colonies (≤ 10 cm) following Hurricanes Irma and Maria, but the trend was not significant at Yawzi Point (*χ* ^2^ = 42.337, *df* = 36, *p* = 0.216) or Tektite (*χ* ^2^ = 43.594, *df* = 36, *p* = 0.180). When years were pooled into “before” and “after” hurricanes, the distribution of colonies among size classes still did vary between periods at Yawzi Point (*χ* ^2^ = 10.591, *df* = 6, *p* = 0.102), but it did at Tektite (*χ* ^2^ = 16.190, *df* = 6, *p* = 0.013). The variation in colony size class structure at Tektite reflected changes following the hurricanes causing an under-abundance of colonies between 10.1 and 20 cm high, and an over-abundance of colonies between 40.1 and 50 cm high (Fig. 3).

**Figure 4:**
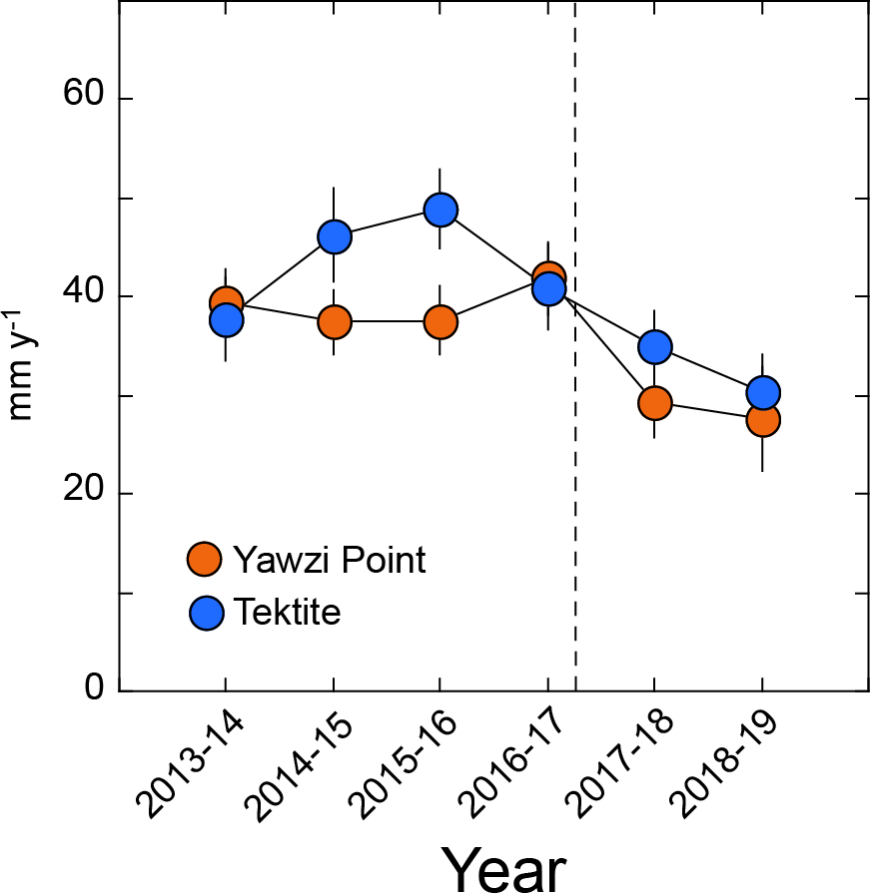
Mean growth rate (±SE) of *Gorgonia ventalina* at Yawzi Point (*n*= 30–63) and Tektite (*n*= 23–31) from 2013–2019. Values based on colonies that were measured in two consecutive years and either did not change in height, or increased in height (i.e., increments of ≥ 0 mm y^−1^). Vertical dashed line shows the impacts of Hurricanes Irma and Maria.

*G. ventalina* for which growth was measured displayed null or positive increments over the year-long intervals, and this criterion was satisfied by 27–63 colonies year^−1^ at Yawzi Point, and 24–34 colonies year^−1^ at Tektite. At Yawzi Point, mean growth varied from 28 ± 5 mm y^−1^ (2018–2019, *n* = 30) to 42 ± 4 mm y^−1^ (2016–2017, *n* = 63), and at Tektite, from 30 ± 4 mm y^−1^ (2018–2019, *n* = 23) to 49 ± 4 mm y^−1^ (2015–2016, *n* = 28) (Fig. 4). Growth at Yawzi Point did not differ among years (*H* = 9.852, *df* = 5, *p* = 0.080), but it was 30% lower in the two years after the hurricanes (28 ± 4 mm y^−1^, *n* = 59) than in the four years before (40 ± 2 mm y^−1^, *n* = 218) (*U* = 7,625, *df* = 1, *p* = 0.030). Growth at Tektite did not differ among the six sampling years (*H* = 10.529, *df* = 5, *p* = 0.062), but was 23% lower in the two years after the hurricanes (33 ± 3 mm y^−1^, *n* = 51) than in the four years before (43 ± 2 mm y^−1^, *n* = 117) (*U* = 3,655, *df* = 1, *p* = 0.020).

### Density-associated effects

**Figure 5:**
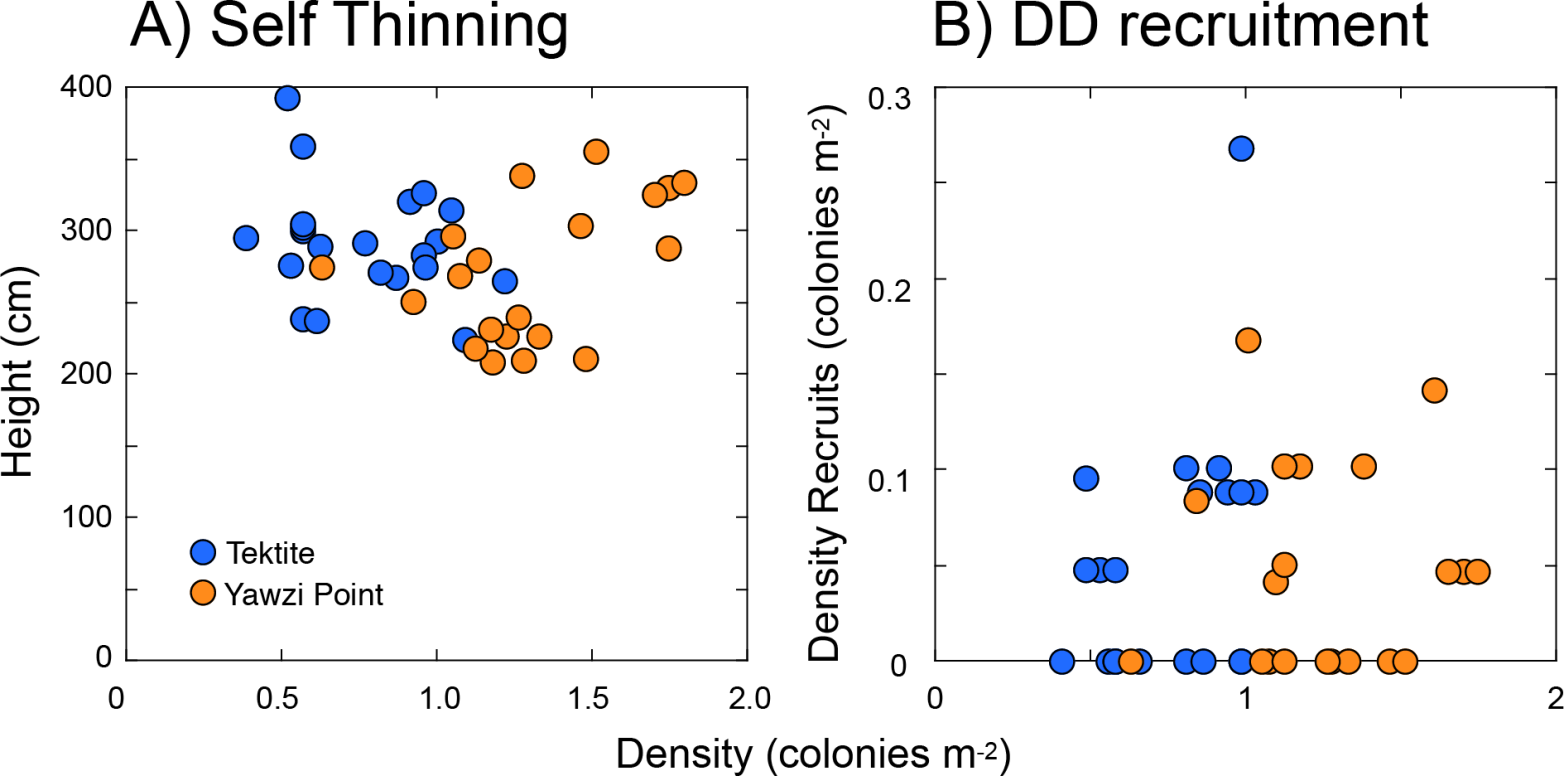
Scatter plots showing features of *Gorgonia ventalina* populations at Yawzi Point and Tektite as tests for density dependent effects. (A) Height versus density as a test for self-thinning, and (B) density of recruits (≤ 5-cm tall) versus the density of larger colonies (> 5-cm tall) as a test for density dependent (DD) recruitment. Points represent one datum y^−1^ from 2013–2019. Neither height versus density (A), nor density of recruits versus density of larger colonies (B), are significantly associated (*p* ≥ 0.050).

Two forms of density-associated effects were explored, one characterizing the relationship between the height and density of colonies (i.e., self-thinning), and the other between the density of recruits and colonies >five cm tall. Using the three transects at each site as replicates (and pooling between sites), the scatterplot of mean colony height and colony density (Fig. 5A) showed no relationship between the two on either linear (*r* =  − 0.062, *df* = 39, *p* = 0.699 [Fig. 5A]) or logarithmic axes (*r* =  − 0.151, *df* = 39, *p* = 0.346). Likewise, there was no relationship between the density of recruits and colonies >five cm tall (*r* = 0.113, *df* = 39, *p* = 0.481 [Fig. 5B]), or between per-capita recruitment and the density of colonies >five cm tall (*r* =  − 0.126, *df* = 39, *p* = 0.432).

## Discussion

Through the lens of scleractinians, the last 40 years has been a sequence of disturbances serving as ecological ratchets promoting low coral cover (Birkeland, 2004; Birkeland, 2019), coral rarity (Edmunds, 2018), and coral extinction (Carpenter et al., 2008). These trends are striking in the Caribbean (Gardner et al., 2003; Jackson et al., 2014), where many reefs are unrecognizable compared to the 1960s (e.g., Goreau, 1959; Jackson et al., 2014; Dustan & Lang, 2019). On some reefs, however, arborescent octocorals have emerged as a dominant taxon (Ruzicka et al., 2013; Lenz et al., 2015) that contributes three-dimensional structure as flexible “forests” with a canopy of closely located branches (Rossi et al., 2017; Tsounis, Steele & Edmunds, 2020). The implications of this transition depend on the longevity of the new assemblages, some of which have been resilient to disturbances over decades (Tsounis & Edmunds, 2017), and have persisted through major hurricanes (Lasker et al., 2020). The present study shows that stands of *Gorgonia ventalina* on shallow reefs have maintained high densities over 7 years that included two large hurricanes in September 2017 (Edmunds, 2019a; Edmunds, 2019b). Bigger colonies were more abundant (and smaller colonies less abundant) after the storms than before, and growth rates declined following the storms, but these effects did not statistically affect density. They unfolded at mean densities of <1.8 colonies m^−2^, at which there was no evidence of density-dependence in the form of self-thinning or variable recruitment. As an iconic member of the octocoral forests that are becoming more prominent on Caribbean reefs, the resilience of *G. ventalina* to storms indicates that this species will play an important role on future reefs that are likely to have a greater abundance of octocorals.

The densities of *G. ventalina* at Yawzi Point and Tektite (0.7–1.5 colonies m^−2^) are high compared to nearby sites that have been surveyed from 2014–2019 (i.e., 0.4–1.3 colonies m^−2^) (Lasker, 2019), Jamaica (Discovery Bay) in the late 1960s (*Gorgonia* sp. 0.02–0.21 colonies m^−2^ in the lagoon, and 0–0.09 colonies m^−2^ at ∼10–15 m depth on the fore reef (Kinzie, 1973)), the Florida Keys from 1997–2003 (<8-m depth, 0.86–1.11 colonies m^−2^(Kim & Harvell, 2004)), and northwest Cuba from 2008–2015 (10-m depth, 0–2.10 colonies m^−2^, region-wide average 0.39 colonies m^−2^(Rey-Villiers et al., 2020)). All these densities are low compared to the values reported for *G. ventalina* from Puerto Rico in 1986 (7–11-m depth, 3.5–5.9 colonies m^−2^at two sites (Yoshioka & Yoshioka, 1989)), and Panama in 1971 (6.2 colonies m^−2^ (Birkeland, 1974)).

The densities of *G. ventalina* recruits at Yawzi Point and Tektite (0–0.12 colonies m^−2^) are low compared to previous studies, including from 7-m depth off Puerto Rico (0.25, 1.80 and 0.28 colonies m^−2^ in spring 1984, spring 1985, and spring 1988, respectively (Yoshioka, 1996)), and 2-m depth off Panama over 1971-1972 (0.15–0.40 colonies m^−2^ (Birkeland, 1974)). In the Florida Keys, *G. ventalina* recruited at <0.12 colonies m^−2^ prior to 2002 (colonies <10-cm tall at <8-m depth), but at 0.18–0.22 colonies m^−2^ from 2002–2003 (Kim & Harvell, 2004). As recruitment (colonies <4-cm tall) in a subset (30–50% of the same plots) of the present areas was 0.20–0.35 colonies m^−2^ in 2013 and 2014 (Privitera-Johnson, Lenz & Edmunds, 2015), spatial variation in recruitment of *G. ventalina* indicates caution is required in concluding that recruitment is low in St. John compared to other locations. The mean heights of *G. ventalina* at Yawzi Point and Tektite (24.4–40 cm) are comparable to heights previously reported from Puerto Rico (7–11-m depth, mean of 24.0–33.7 cm (Yoshioka & Yoshioka, 1991)), Panama (∼2 m depth, mean of 36–88 cm (Birkeland, 1974)), and the Florida Keys (≤ 8-m depth, median of 26–40 cm (Kim & Harvell, 2004)). Finally, the growth rates of *G. ventalina* in St. John (2.8–4.9 cm y^−1^) compare to growth rates of 1.9–2.3 cm y^−1^ in Puerto Rico from 1983–1988 (7–11 m depth (Yoshioka & Yoshioka, 1991)), 2.6 cm (240 d)^−1^ in Panama (∼2-m depth (Birkeland, 1974)), 3.9 cm y^−1^ in Puerto Rico from 2003–2006 (2–25-m depth (Borgstein, Beltrán & Prada, 2020)), and 4.9 ± 1.2 cm y^−1^ on the shallow reefs of Dry Tortugas (1910–1911 (Cary, 1914)). Together, the aforementioned contrasts indicate that the stands of *G. ventalina* at Yawzi Point and Tektite from 2013–2019 consisted of unusually high densities of averaged-sized colonies that grew at regionally-representative growth rates, but may not have been well supported by recruitment (i.e., colonies ≤ 5-cm tall).

From 2013 to 2019, the densities and sizes of *G. ventalina* at Yawzi Point and Tektite were similar among years, even though the study was interrupted by two Category 5 hurricanes in September 2017. Both storms passed ≤ 86 km from St. John with wind speeds of 266–298 km h^−1^, wave heights of 5.6–7.9 m (8 km from the study sites), and 11–17 cm of rain on the day of impact (Edmunds, 2019a). While damage underwater qualitatively was conspicuous (Edmunds, 2019a; Edmunds, 2019b; Gochfeld et al., 2020), the quantitative impacts were modest for scleractinians at 7–14-m depth (Edmunds, 2019a; Edmunds, 2019b), octocorals at 6–9-m depth (Lasker et al., 2020), and around St. Thomas, also for sponges (Gochfeld et al., 2020). Nevertheless, ∼5,852 *Gorgonia* spp. colonies were estimated to have washed onto the beach of Great Lameshur Bay in November 2017, whereas ∼61 were found in June 2017, and potentially the colonies in November 2017 came from the adjacent reefs. This inference is supported by the non-significant declines in density of *G. ventalina* between August 2017 and August 2018, representing a reduction of 0.20 colonies m^−2^ at Yawzi Point and 0.19 colonies m^−2^ at Tektite (Fig. 2). With fringing reefs ∼75 m wide along about half of the western coast of Cabritte Horn (690 m) and half the eastern coast of Yawzi Point (298 m), these declines in density of *G. ventalina* could have supplied ∼14,820 detached colonies, which is more than double what was estimated to be on the beach in Great Lameshur Bay in November 2017.

Previous studies of the response of *Gorgonia* spp. to storms have shown that colonies resist breakage, but are removed through failure of the substratum to which their holdfasts are attached (Kinzie, 1973; Birkeland, 1974; Yoshioka & Yoshioka, 1991; Zuluaga-Montero & Sabat, 2012). Without major disturbances, *G. ventalina* has high annual survivorship (∼92–95% (Birkeland, 1974); Yoshioka & Yoshioka, 1991), but over the last 25 years, mortality has been accentuated by the disease Aspergillosus, which killed 39% of sea fans (>20-cm tall) in the Florida Keys between 1997 and 2003 (Kim & Harvell, 2004). While Aspergillosus was seen on *G. ventalina* during the present study, the rarity of cases and the sustained population size (even with low recruitment) suggested this was not an ecologically important disease in this location between 2013 and 2019 (cf. Kim & Harvell, 2004). In St. John, Hurricanes Irma and Maria slightly reduced the density of *G. ventalina*, in part through the loss of large colonies, but mostly through the removal of small- (≤ 10-cm tall) and intermediate- (>10 and ≤ 40-cm tall) sized colonies. Presumably, these smaller colonies were weakly attached to the reef, and hence more readily removed by waves or sediment scour (Yoshioka & Yoshioka, 1987; Yoshioka, 2009). Since both recruitment and growth rates were depressed in the two years following the hurricanes, it probably was the removal of the smaller colonies that led to an increase in mean colony size. While the hurricanes also increased the number of ragged colonies in 2018 and 2019 versus the previous 5 years (20% versus 7% at Yawzi Point, and 16% versus 10% at Tektite, respectively), these events did not greatly alter colony height and, therefore, are unlikely to have mediated change in mean colony height at each site.

By focusing on sites where *G. ventalina* accounted for 61–66% of arborescent octocorals, tests for density-associated phenomena were less likely to be confounded by other species of octocorals. For self-thinning and recruitment, there was no evidence of density-association for these phenomena, although this outcome must be interpreted within the limitations of the study, including the range of densities that were evaluated, and the sample size employed (number of transects). Caution is also warranted in interpreting these trends because they were obtained by pooling results among years, which relies on the untested assumption that the demographic state variables were independent among years for common transects.

Self-thinning is well known in terrestrial forests (Westoby, 1981; Westoby, 1984), and it describes the reduction in density of even-aged trees that mediates an increase in size of the remaining trees, with this effect most strongly expressed at saturating biomass (Westoby, 1984; Weller, 1987). On a double logarithmic plot of size (i.e., biomass) against density, the slope commonly is considered to be -1.5 in the presence of self-thinning (White, 1981; Westoby, 1984). Density-dependent recruitment is well known in the marine environment (Caley et al., 1996; Hixon, Pacala & Sandin, 2002), but density-associated recruitment is only diagnostic of density-dependent recruitment if per capita recruitment covaries with adult density. While density-dependent larval supply is not possible in demographically open populations like those of mass spawning taxa, including *G. ventalina*, density-dependent settlement and recruitment could occur if larval delivery to the benthos is modified by flow effects within octocoral forests (Guizien & Ghisalberti, 2016), or if recruitment is modified by proximity to adults (Marhaver et al., 2013). Evidence of density-dependent effects were not detected in the present study, even though density-associated recruitment was reported from this locality for *G. ventalina* over 2013 and 2014 (Privitera-Johnson, Lenz & Edmunds, 2015), and *Eunicea* spp. in 3 of 4 years from 2014–2017 (Edmunds & Lasker, 2019), and signs of self-thinning were found for *Eunicea* spp. in shallow water (9-m depth) adjacent to Tektite (Edmunds & Lasker, 2019). Self-thinning also has been detected in the Mediterranean octocoral *Paramuricea clavata* (Linares et al., 2008).

The absence of support for self-thinning in *Gorgonia ventalina* is consistent with the mostly null results of testing for this effect in St. John (Edmunds & Lasker, 2019), but in the present analysis there is added weight to the outcome because the study system more closely approximated the monospecific, even-aged stands to which self-thinning is best applied (Weller, 1987; Norberg, 1988). Together, the weight of the evidence remains against self-thinning as an important ecological mechanism structuring octocoral forests on the shallow reefs of St. John (Edmunds & Lasker, 2019). Even though the present stands of *G. ventalina* occurred at high densities relative to most adjacent areas, the discrepancy in density versus what has been reported for this species (5.9–6.2 colonies m^−2^ (Birkeland, 1974; Privitera-Johnson, Lenz & Edmunds, 2015)) and all species combined (76.1 colonies m^−2^ (Yoshioka & Yoshioka, 1989)) leaves open the possibility that self-thinning may occur in a “crowded” octocoral community (Westoby, 1984; Weller, 1987).

For the positive density-associated recruitment detected for *Gorgonia ventalina* in St. John in 2013 and 2014, Privitera-Johnson, Lenz & Edmunds (2015) used results from surveys at the present sites augmented with 6–8 sites along the south coast. This collection of sites provided a range of densities with an upper value (5.9 colonies m^−2^) that was 3.3-folder greater than the highest value recorded in the present study (1.8 colonies m^−2^ along one transect at Yawzi Point in 2017), which raises the possibility that the null results for density-association of recruitment reflects the narrower range of densities of *G. ventalina* that were included in the present analysis. Although cause-and effect was not established in Privitera-Johnson, Lenz & Edmunds (2015), the positive density-association was hypothesized to result from several possibilities including self-recruitment, larval entrainment, enhanced fertilization, or enhanced post-settlement success. Quantitative manipulations of octocoral densities rarely have been completed, but two examples produced contradictory results: Birkeland (1974) removed 83% of the colonies of *G. ventalina* from a 20 m^2^ area in shallow water in Panama in 1971 (original density = 6.2 colonies m^−2^), and recruitment was elevated by ∼100%. In contrast, Yoshioka (1996) removed octocorals (all species) from eight small quadrats (0.5 m^2^, original densities 42-63 colonies m^−2^) at 7–11 m depth of Puerto Rico in 1987, and found recruitment was reduced by 15–94% over 1–5 months. Together, the present and previous results are consistent with the possibility that the relationship between recruitment and adult density in *G. ventalina* is driven by multiple phenomena, possibly with different mechanisms operating at different densities. For example, larval entrainment within canopies might function at low-to-medium densities, space limitation might operate at high densities, and enhanced fertilization might operate as extreme high densities.

In summary, the present results describe 7 years of population resilience for *G. ventalina* on the shallow reefs of St. John, even with respect to the effects of two Category 5 hurricanes. It is reasonable to infer, therefore, that *G. ventalina* will remain a persistent member of the octocoral forests that are emerging on present-day Caribbean reefs. While more work is required to address density-dependent demographic effects in this species, notably involving manipulative experiments and greater replication, the evidence presented here is equivocal with regards to the role of density-associated recruitment and self-thinning in modulating population size of *G. ventalina*.

##  Supplemental Information

10.7717/peerj.10315/supp-1 Supplemental Information 1 Heights of Gorgonia that are used to support all analyses in the paper The height of all colonies along three transects that were surveyed art Yawzi Point and Tektite from 2013 to 2019. They can be used to calculate mean density (using transects as replicates), mean heights (using colonies as replicates) and mean growth (using colonies that were relocated in consecutive years).

## Additional Information and Declarations

### Field Study Permissions

The following information was supplied relating to field study approvals (i.e., approving body and any reference numbers): Virgin Islands National Park approved the field study (VIIS-2013-SCI-0015).

### Data Availability

The following information was supplied regarding data availability: The raw data is available in the Supplemental File and at BCO-DMO (https://www.bco-dmo.org/): DOI: 10.26008/1912/bco-dmo.827851.1 and 10.26008/1912/bco-dmo.827897.1.
